# Contributions of Clinical Simulation to Group Cohesion: A Quasi-Experimental Study

**DOI:** 10.3390/ejihpe16020029

**Published:** 2026-02-16

**Authors:** José Manuel García-Álvarez, Alfonso García-Sánchez, José Luis Díaz-Agea

**Affiliations:** 1 Health Sciences PhD Program, Catholic University of Murcia (UCAM), Campus de los Jerónimos nº 135, Guadalupe, 30107 Murcia, Spain; 2 Faculty of Nursing, Catholic University of Murcia (UCAM), Campus de los Jerónimos nº 135, Guadalupe, 30107 Murcia, Spain; agarcia@ucam.edu; 3 Faculty of Nursing, University of Murcia, Campus de Ciencias de la Salud, Edificio LAIB/DEPARTAMENTAL, El Palmar-Murcia, 30120 Murcia, Spain; agea@um.es

**Keywords:** group cohesion, simulated learning environment, nursing

## Abstract

(1) Background: The complexity of today’s healthcare system requires the formation of highly cohesive work teams that guarantee safe and high-quality care. Clinical simulation has become established as a pedagogical strategy capable of promoting the collaborative skills of teams of students and healthcare professionals. The objective of this study was to analyze the influence of learning through clinical simulation on group cohesion in nursing student teams. (2) Methods: A pre–post quasi-experimental study without a control group was conducted with final-year nursing students using the short Spanish version of the Group Environment Questionnaire, validated for nursing students. This questionnaire was administered twice, before and after participation in clinical simulation sessions. (3) Results: Clinical simulation significantly increased group cohesion in most items and in all dimensions with moderate to large effect sizes (r > 0.5). The Group Integration-Task (GI-T) dimension showed the greatest improvement after clinical simulation. Although causal relationships cannot be established, the results suggest an association between exposure to clinical simulation and increased group cohesion. (4) Conclusions: Clinical simulation was associated with significant improvements in both task-oriented and social dimensions of group cohesion among nursing students. These findings suggest that clinical simulation may enhance collaboration, communication, and commitment to shared goals within student teams. Future studies including control groups are needed to confirm these associations and further explore the impact of clinical simulation on team performance in both student and healthcare professional contexts.

## 1. Introduction

High levels of technology development and the need for continuity of care are the determining factors in healthcare today. This increase in healthcare complexity means that individual work is no longer sufficient to meet all needs and requires healthcare professionals to work as a team in order to provide efficient, high-quality care to patients (Braš et al., 2022; Salihović et al., 2024). Teamwork allows for more efficient use of available healthcare resources while simultaneously reducing stress and increasing satisfaction among healthcare professionals (Anselmann et al., 2023; Borrego et al., 2012; Schmutz et al., 2019; Wang et al., 2023).

To work effectively as a team, healthcare professionals need to develop a set of non-technical skills, including effective communication, coordination, complementarity, trust, and commitment. In this context, “technical” and “non-technical” skills are used rather than “hard” and “soft” skills. These terms are widely used in the healthcare simulation literature and are considered more precise for the competencies targeted in this study. Technical skills refer to task-specific competencies, such as performing clinical procedures, whereas non-technical skills encompass interpersonal, cognitive, and teamwork abilities that support safe and effective performance. These non-technical skills are essential for strengthening the cohesion of the healthcare team, enabling the achievement of common goals within a work environment characterized by excessive workload and high emotional involvement from both patients and professionals (Anselmann et al., 2023; Stahel et al., 2022; Zeng et al., 2022).

Group cohesion refers to the process that keeps a healthcare team united as it works toward achieving common care goals. Adequate cohesion within healthcare teams ensures comprehensive patient care and also promotes the emotional well-being of healthcare professionals. Two aspects can be distinguished within group cohesion: task cohesion, or commitment to achieving common goals, and social cohesion, or the degree of interaction among team members. Addressing these two aspects, both as a group and individually, will give rise to the four dimensions that make up group cohesion: Group Integration-Task (GI-T) or degree of group union to achieve common objectives, Group Integration-Social (GI-S) or degree of group union to develop social relations within the group, Individual Attractions to Group-Task (ATG-T) or individual motivations towards common objectives, and Individual Attractions to Group-Social (ATG-S) or individual motivations towards social relations within the group (Carron et al., 1985; Gu & Xue, 2022; Mehdi, 2023; Sghari et al., 2019).

The Group Environment Questionnaire (GEQ) allows for the assessment of these four dimensions of group cohesion (Carron et al., 1985; Eys et al., 2007). Among the various adaptations and validations of the original questionnaire for the Spanish language and context (Checa & Bohórquez, 2020; Leo et al., 2015) is the short version validated for nursing students undergoing clinical simulation training (García-Álvarez et al., 2025). This version has shown acceptable values for internal consistency, homogeneity, and test–retest reliability, both for the questionnaire as a whole and for its individual dimensions. Exploratory and confirmatory factor analyses of this version have confirmed that it adequately fits the original four-dimensional model of group cohesion (García-Álvarez et al., 2025). Therefore, the short Spanish version of the Group Environment Questionnaire (GEQ) can be considered a valid and reliable instrument for analyzing group cohesion in nursing student teams undergoing training in simulated learning environments (García-Álvarez et al., 2025).

In this context, clinical simulation emerges as a particularly suitable educational strategy for fostering the competencies underlying group cohesion.

Clinical simulation has demonstrated its ability to develop non-technical skills considered essential for improving teamwork and fostering group cohesion among teams of students and healthcare professionals. Simulated scenarios replicate realistic clinical situations that students address collaboratively, promoting learning, skill development, and enhanced teamwork (Allard et al., 2020; Flynn et al., 2022; Griffin et al., 2020; Peddle, 2019).

Each simulation session begins with a prebriefing phase in which learning objectives and team roles are defined. This is followed by an active scenario phase in which students perform patient care tasks under realistic time and resource constraints. Finally, a debriefing phase allows students to reflect on their actions and consolidate their learning (Alonso-Peña & Álvarez Álvarez, 2023; Halamek et al., 2019).

Through repeated cycles of simulation and guided reflection, students practice both technical and non-technical skills, experience shared challenges, and develop situational awareness, trust, and coordination within the team (Allard et al., 2020; Griffin et al., 2020; Wu, 2025). Clinical simulation promotes situational awareness, efficient use of available resources, strengthened decision-making processes, and effective information transfer. In addition, it leads to increased trust and mutual respect, greater commitment and collaboration, appropriate distribution of leadership, and substantial improvement in stress management (Allard et al., 2020; Flynn et al., 2022; Griffin et al., 2020; Innocenti et al., 2022; Peddle, 2019; Lee & Lee, 2022; Lynch, 2020; Ounounou et al., 2019; Wu, 2025).

Analyzing the influence of clinical simulation on group cohesion using this questionnaire would allow for the development of intervention programs specifically designed to strengthen group cohesion within healthcare teams. Increased group cohesion among healthcare teams would improve the efficiency and quality of patient care. In addition, it would reduce stress and increase the job satisfaction of healthcare professionals.

The objective of this study was to analyze the influence of learning through clinical simulation on group cohesion in nursing student teams.

## 2. Materials and Methods

### 2.1. Ethics Statement

This study was conducted in accordance with the ethical principles established by the Declaration of Helsinki (World Medical Association, 2013) and with the approval of the Ethics Committee of the Catholic University of Murcia (UCAM).

All participating students were fully informed about the characteristics of the study, emphasizing the voluntary nature of their participation and their right to withdraw at any time. Written informed consent was obtained from all participants, and confidentiality was ensured by the absence of any identifying information.

### 2.2. Study Design

To analyze the changes in group cohesion produced by clinical simulation sessions, a quasi-experimental study with a pre–post design without a control group was conducted, following the TREND (Transparent Reporting of Evaluations with Non-Randomized Designs) checklist for non-randomized intervention studies (Des Jarlais et al., 2004).

### 2.3. Subjects and Scope of Study

The study participants were fourth-year nursing students from the Catholic University of San Antonio of Murcia, Spain (UCAM) and the University of Murcia, Spain (UMU) who had participated in clinical simulation sessions between October 2023 and July 2024. These students were selected for this study because they had prior experience in training in simulated environments. This prior experience reduced the variability among the participating students and allowed the observed results to be attributed with a high probability to the intervention performed.

### 2.4. Sample Selection

The inclusion criteria were: being a fourth-year nursing degree student at participating universities, having completed all clinical simulation sessions, and wanting to participate voluntarily in the study.

The sample was selected using non-probability convenience sampling based on the groups assigned by the Nursing Practice Unit of each of the participating universities.

### 2.5. Measurement Instrument and Data Collection

To collect the information, each participant completed the same questionnaire twice: once before the first simulation session (pre-test) and again after completion of the final simulation session (post-test).

Between the two rounds of questionnaires, four four-hour simulation sessions were conducted, one per week, with stable groups of two to three students. Each group prepared one scenario per session, allowing the practice of complex technical and non-technical skills in the field of special care. The simulations were carried out using a high-fidelity simulator (SimMan 3G; Laerdal, Stavanger, Norway). After each simulated scenario, a structured Plus/Delta debriefing was conducted, designed to identify the aspects that should be maintained (Plus) and those that require improvement (Delta).

The initial questionnaire included a section with sociodemographic data and another with the short Spanish version of the Group Environment Questionnaire (GEQ), validated for nursing students participating in clinical simulation practices (García-Álvarez et al., 2025). The final questionnaire included only the GEQ. The GEQ consists of 12 items with a five-point Likert-type response format, ranging from strongly disagree (1) to strongly agree (5). Items 1, 3 and 5 assessed the dimension Individual Attractions to Group-Social (ATG-S); items 2, 4 and 6 analyzed the dimension Individual Attractions to Group-Task (ATG-T); items 7, 9 and 11 assessed the dimension Group Integration-Social (GI-S); and items 8, 10 and 12 analyzed the dimension Group Integration-Task (GI-T) (Table 1) (García-Álvarez et al., 2025).

The variables analyzed were: university of origin, age, gender, region of origin, previous academic qualification, work activity, scores of the questionnaire items, scores of the questionnaire dimensions, and total questionnaire score.

### 2.6. Data Analysis

The information was collected in a database that was later analyzed using the SPSS v26^®^ statistical software for Windows (Armonk, NY, USA: IBM Corp.).

For dichotomous or polytomous qualitative variables, frequencies and percentages were calculated. For quantitative variables, the mean was calculated as a measure of central tendency and the standard deviation as a measure of dispersion, verifying their normality using the Kolmogorov–Smirnov test. For ordinal qualitative variables, the median was calculated as a measure of central tendency and the interquartile range (IQR) as a measure of dispersion (Kaliyadan & Kulkarni, 2019; Mishra et al., 2018). Inferential statistical analysis was also performed using appropriate tests based on the characteristics of the variables analyzed (Table 2) (Patel, 2021a, 2021b).

To assess the influence of clinical simulation on group cohesion, variations in item and dimension scores were analyzed between the two administrations of the GEQ, before and after clinical simulation sessions. Given the ordinal nature of the questionnaire items and the non-normal distribution of the data, non-parametric statistical analyses were performed. Pre- and post-intervention scores were compared using the Wilcoxon signed-rank test for related samples at the individual level. Although students participated in simulation activities organized in teams, the analysis was conducted at the individual level because group cohesion was assessed through individual perceptions, as captured by the questionnaire. In addition, complete information regarding stable team membership across all simulation sessions was not available, which precluded the use of multilevel or mixed-effects models. This approach was adopted to avoid imposing unverifiable assumptions about the dependency structure of the data (McNabb & Murayama, 2021; Rosner et al., 2006).

The effect size of clinical simulation practices on group cohesion was estimated using Rosenthal’s r, calculated as *r* = |Z|/√N, where Z is the standardized test statistic from the Wilcoxon signed-rank test and N is the number of valid paired observations. Values below 0.3 were considered small effects, values between 0.3 and 0.49 were considered medium effects, values between 0.5 and 0.69 were considered large effects, and values equal to or greater than 0.7 were considered very large effects (Fritz et al., 2012).

## 3. Results

The study sample consisted of 188 students from UCAM and 123 students from UMU. Most of the students were between 21 and 22 years old (63.65%), were female (78.14%), and came from the region where the universities were located, the Region of Murcia (Spain) (71.06%). A significant percentage of the students did not have a previous degree (81.68%) and were not working (91.00%).

Statistically significant associations were found between age and gender (*p* = 0.001), previous academic qualification (*p* = 0.000), and work activity (*p* = 0.000). Significant associations were also observed between region of origin and university (*p* = 0.000) and between work activity and previous academic qualification (*p* = 0.000).

Following the clinical simulation sessions, overall group cohesion increased, with higher median scores across most items and all dimensions in the post-simulation questionnaire. Several items presented high baseline medians, suggesting a potential ceiling effect that may have limited the magnitude of change. While item-level dispersion remained stable, the reduction in IQR values at the dimension level indicates greater consensus among participants’ responses (Table 3 and Table 4).

Although statistically significant differences were observed for all items, effect sizes ranged from small to large and were generally lower in items with higher baseline scores, indicating limited room for improvement. The largest effects were observed at the dimension and total score levels, particularly in the GI-T dimension, while social dimensions showed slightly smaller gains overall (Table 5 and Table 6). In line with this, the total group cohesion score and the GI-T dimension exhibited the greatest increases following clinical simulation. The variability in effect sizes across items suggests that the impact of the intervention was not uniform across all aspects of group cohesion. These findings indicate associations consistent with exposure to clinical simulation rather than definitive evidence of causality.

## 4. Discussion

The superior scores on the items and dimensions of the second questionnaire indicate that clinical simulation was associated with higher group cohesion among nursing students, consistent with prior evidence on simulation improving teamwork and non-technical skills (Allard et al., 2020; Flynn et al., 2022; Griffin et al., 2020; Lee & Lee, 2022; Lynch, 2020; Peddle, 2019). Effect sizes were large for all dimensions of the GEQ, although individual item effect sizes varied, reflecting the heterogeneous sensitivity of different aspects of group cohesion.

The results obtained in this research coincide with previous studies that also observed that carrying out simulation activities in sports or educational contexts favored the formation of work teams by increasing group cohesion, supporting the use of simulation as an effective strategy to promote team cohesion in different training areas (Agbo et al., 2020; Kwon, 2024; Melo & Cole, 2024; Roh et al., 2024; Stewart et al., 2016).

The group cohesion dimension GI-T showed the greatest improvement after the clinical simulation sessions. This suggests that simulation practices can be particularly effective in strengthening bonds related to achieving shared goals, promoting collaboration, coordination, and collective problem-solving. Clinical simulation fosters essential non-technical skills that support the achievement of shared objectives and enhance group cohesion more effectively than case-based learning (Griffin et al., 2020; Wu, 2025). By engaging in collaborative problem-solving and navigating challenges together, team members strengthen both emotional and professional bonds, promoting a heightened sense of belonging and mutual support within the group (Agbo et al., 2020; Fornander et al., 2024; Melo & Cole, 2024; Mende et al., 2020; Power et al., 2022; Roqueta-Vall-Llosera et al., 2024; Stewart et al., 2016).

These non-technical skills are essential for achieving common objectives and are recognized for their ability to increase group cohesion more effectively than case-based or purely theoretical learning (Griffin et al., 2020; Wu, 2025). Clinical simulation is a shared experience carried out through teamwork, which helps to increase the sense of belonging to the team. This learning tool allows team members to face challenges, successes, and failures together. All these non-technical skills fostered by clinical simulation strengthen the emotional and professional bond between team members, thus promoting group cohesion (Fritz et al., 2012; Stewart et al., 2016; Lafferty et al., 2016; Mathieu et al., 2015; Mende et al., 2020; Power et al., 2022; Roqueta-Vall-Llosera et al., 2024; Fornander et al., 2024; Roh et al., 2024).

The observed improvement in the ATG-S dimension following clinical simulation sessions suggests a potential increase in participants’ desire to belong to the team, an interest that goes beyond mere commitment to achieving common goals. This finding appears to reflect a strengthening of collective identity, interpersonal trust, and perceptions of mutual support. Enhancing this sense of belonging could support motivation, team stability, and the willingness to collaborate proactively in both simulated and real healthcare settings, aspects that may be associated with greater group cohesion (Benchadlia et al., 2023; Mabry et al., 2020; Melo & Cole, 2024; Roh et al., 2024).

The increase in the GI-S dimension has highlighted the team members’ commitment to establishing interpersonal connections outside of clinical simulation practices. This result suggests that the shared experiences during simulation have not only strengthened collaboration within the training environment but have also fostered the creation of stronger and more lasting personal relationships. These bonds established outside of academic activities can contribute to improving the climate of trust, open communication, and mutual support, factors that will allow for a much more cohesive and effective work dynamic when team members face future simulated or real clinical situations (Benchadlia et al., 2023; Mabry et al., 2020; Melo & Cole, 2024; Roh et al., 2024).

Finally, the improvement in the ATG-T dimension suggests that clinical simulation promotes the prioritization of group objectives over individual interests. This result indicates that clinical simulation functioned as a collective experience in which the competencies of the team as a whole appeared to exceed the mere sum of its members’ individual competencies, prioritizing the collective over the individual and potentially contributing to the strengthening of teamwork and group cohesion (Benchadlia et al., 2023; Mabry et al., 2020; Melo & Cole, 2024; Roh et al., 2024).

In the context of this study, clinical simulation appears to have contributed to the development of effective team communication, a key factor for group cohesion. Simulation-based training has been associated with improvements in clear communication and conflict management, which may enhance the quality of team interactions, foster mutual trust, and potentially contribute to greater group cohesion (Allard et al., 2020; Flynn et al., 2022; Griffin et al., 2020; Peddle, 2019; Peng et al., 2019; Robson et al., 2023).

Clinical simulation also provides an excellent opportunity to learn how to address potential conflicts that may arise within a team, helping to improve trust and interpersonal relationships among its members. By recreating complex situations in a safe environment, participants can practice conflict management strategies, such as assertive communication, active listening, and collaborative problem-solving, without the risks associated with a real clinical setting. This training promotes the early identification of tensions, the collaborative resolution of disagreements, and the strengthening of mutual respect. As a result, teams acquire a greater capacity to face challenges, maintain cohesion, and work more harmoniously in demanding contexts such as the healthcare setting (Barr et al., 2020; Gunasingha et al., 2023; Johnston & Pierce, 2023).

Clinical simulation is primarily based on collaborative learning and group reflection. These characteristics enable clinical simulation to foster a culture of mutual support within an inclusive environment, where each team member feels valued regardless of their hierarchical level or experience. The opportunity to share perceptions, emotions, and analysis during debriefing encourages all participants to recognize themselves as active members of the improvement process, thus reinforcing equal participation and respectful listening. This inclusive environment allows errors made during clinical simulation practice to be viewed as opportunities for improvement rather than sources of blame. By addressing failures from a growth perspective, participants develop greater openness to feedback and a more proactive attitude toward self-reflection and continuous improvement. This constructive view of mistakes strengthens the sense of collective achievement, motivates the team to work better in the future, and ultimately promotes group cohesion by consolidating the idea that progress is the result of shared effort and not isolated individual performances (Cust & Boden, 2022; Power et al., 2022).

In short, clinical simulation increases group cohesion by providing a safe and inclusive space where team members can practice critical skills, face common challenges, and reflect together on any present or future tasks. This protected learning environment allows participants to experiment, make decisions, and manage complex situations without the risk inherent in real practice, promoting greater openness to collaborate, communicate, and learn together. Clinical simulation can improve both the technical and non-technical skills of team members, strengthening interpersonal bonds between them and building more effective, resilient, and cohesive teams. It also promotes understanding of professional roles, increases the ability to anticipate the needs of the group, and fosters a culture of support and shared responsibility. The recurring practice of simulated scenarios improves group work dynamics and promotes team success in real clinical settings, as the lessons learned are naturally transferred to healthcare practice. Clinical simulation allows for the formation of teams that are more confident, coordinated, and better prepared to deal with high-pressure situations more effectively.

Although this study was conducted with nursing students in a simulated environment, the observed improvements in group cohesion have potential implications for patient care. Higher levels of team cohesion are associated with more effective communication, collaboration, and coordination in healthcare teams, which can contribute to higher-quality care and reduced errors. By fostering trust, shared goals, and proactive teamwork behaviors, clinical simulation may help prepare students for safer and more efficient performance in real clinical settings. These implications should be interpreted cautiously, as the transfer of effects from simulation to practice was not directly assessed in this study.

In addition, improved group cohesion may foster a more homogeneous approach to patient care (Anselmann et al., 2023; Schmutz et al., 2019), reducing variability in clinical decisions and enhancing quality and safety. When team members share common goals, roles, and communication strategies, the care provided is more consistent and coordinated, reducing variability in clinical decisions and actions. This consistency can enhance both the clinical quality of care and the quality perceived by patients, as patients experience more predictable, reliable, and coordinated interventions. Although this study was conducted in a simulated educational setting, these findings suggest that strengthening team cohesion could support more uniform and high-quality patient care in real clinical contexts.

Additionally, clinical simulation may help to harmonize students’ knowledge and skills at the beginning of the program, particularly when participants come from different educational backgrounds or prior learning experiences. By providing all students with shared learning objectives, exposure to comparable clinical scenarios, and structured feedback, simulation could contribute to reducing initial variability in understanding and competence (Allard et al., 2020; Flynn et al., 2022). This more homogeneous starting point may facilitate teamwork and the development of group cohesion, as team members begin collaborative activities with more comparable expectations and baseline competencies. However, this potential homogenizing effect was not directly measured in the present study and should be explored in future research.

The selection of participating students through non-randomized convenience sampling and their affiliation with two different universities in the same geographic area may limit the external validity of this study, as the results obtained could be heavily influenced by the specific characteristics of the region where they are located. This implies that the findings may not be directly applicable to nursing students from other universities, regions, or different cultural contexts. To allow for a more robust generalization of the results, it would be advisable to conduct further studies with a more diverse and representative sample, selected through random sampling at universities in different regions. This would also allow for an assessment of whether the effects of clinical simulation on group cohesion persist across different educational settings, strengthening the external validity and practical applicability of the conclusions.

Conducting this study with nursing students rather than practicing professionals could affect the generalizability of the results, as academic activity can differ significantly from professional practice, which is influenced by patient care pressure, case complexity, responsibility, and the dynamics of multidisciplinary teams. Therefore, it would be advisable to conduct future studies assessing group cohesion in real healthcare settings, where teams include physicians, nurses, technicians, and other professionals with varying levels of experience and responsibility. Analyzing group cohesion among the different professionals comprising healthcare teams would allow researchers to identify how interpersonal relationships and collaboration influence teamwork effectiveness, decision-making, and care coordination. Likewise, such studies could provide more direct evidence on the impact of group cohesion on the quality of healthcare, patient safety, and the satisfaction of both healthcare professionals and users, thus providing valuable information for designing interventions aimed at strengthening clinical teams.

Due to the characteristics of the university educational context, it was not possible to establish a control group. All students participated in the same program, where clinical simulation was mandatory and universal, making it impossible to exclude a number of students to establish a control group. Although the pre–post quasi-experimental design without a control group is common in such educational settings, it limits the ability to attribute improvements in group cohesion solely to clinical simulation. Nevertheless, as students engaged exclusively in clinical and simulated practice during the study period, and the key elements of group cohesion—common goals and shared leadership—were present only in the simulations, it is likely that the observed improvements were associated with this intervention. Future studies, including a control group of students not exposed to clinical simulation, would help determine its true effect on group cohesion. Until then, results should be interpreted as associations consistent with exposure to clinical simulation, rather than definitive evidence of causality.

Due to the ordinal nature of the variables and the lack of information on student assignment to the different simulation groups, it was not possible to model the hierarchical structure of the data. Consequently, the Wilcoxon signed-rank test for related samples was selected instead of a hierarchical or mixed-effects model. This conservative approach avoids unverifiable assumptions regarding the dependence of observations, but it limits the ability to analyze variability attributable to group membership. Therefore, the results should be interpreted with caution.

Taken together, these findings highlight the need for further qualitative research to explore in greater depth how clinical simulation contributes to group cohesion among teams of students and healthcare professionals. Such studies would allow for a more nuanced understanding of the subjective experiences, perceptions, and interpersonal dynamics that may influence group cohesion within simulated learning environments. Gaining insight into these factors could help optimize the design and implementation of simulation-based training, ultimately enhancing team functioning and overall performance.

## 5. Conclusions

Clinical simulation was associated with improvements in perceived group cohesion in teams of final-year nursing students across all dimensions analyzed by the short Spanish version of the Group Environment Questionnaire (GEQ) for nursing students.

Group Integration-Task (GI-T) was the dimension that showed the greatest improvement, highlighting the potential of this learning tool to enhance collaboration and commitment among nursing students toward common goals.

These findings provide preliminary evidence that clinical simulation may positively influence perceived group cohesion. Given the methodological limitations related to the lack of a control group, individual-level analysis, and multiple testing, further research using group-level analyses and appropriate controls is needed to confirm these results and evaluate their applicability to professional healthcare teams.

Nevertheless, the observed improvements suggest that regular use of clinical simulation in educational programs may help strengthen teamwork and collaborative skills among nursing students.

## Figures and Tables

**Table 1:** Spanish short version of the Group Environment Questionnaire (GEQ) for nursing students.

Number	Item
1	I like to participate in extracurricular activities with the other members of my group (dinners, excursions …)
2	I am happy with my contributions to the work of the group
3	I have good friends in this group
4	In this group I can perform to the best of my ability
5	Group members are one of the most important social groups to which I belong
6	I like the style of work of this group
7	Group members like to party together
8	Group members join forces to achieve the objectives during the preparation and conduct of the simulation sessions
9	Group members would like to get together a few times after the clinical simulation is over
10	All members take responsibility for a poor group performance
11	Our group members would like to meet in situations other than preparing and conducting simulation sessions
12	If there is a problem during the preparation of the simulation sessions, all members join forces to overcome it

Note: (García-Álvarez et al., 2025).

**Table 2:** Inferential statistics of sociodemographic data.

Variables	Statistical Test
Age and university	Mann–Whitney U
Age and gender	Mann–Whitney U
Age and region of origin	Kruskal–Wallis
Age and previous academic qualification	Mann–Whitney U
Age and work activity	Mann–Whitney U
Gender and university	Chi-square
Gender and region of origin	Chi-square
Gender and previous academic qualification	Chi-square
Gender and work activity	Chi-square
Region of origin and university	Chi-square
Region of origin and previous academic qualification	Chi-square
Region of origin and work activity	Chi-square
University and previous academic qualification	Chi-square
University and work activity	Chi-square
Work activity and previous academic qualification	Chi-square

**Table 3:** Descriptive statistics of the questionnaire items.

Initial questionnaire	Item 1	Item 2	Item 3	Item 4	Item 5	Item 6	Item 7	Item 8	Item 9	Item 10	Item 11	Item 12
Median	4	4	4	4	3	4	3	4	4	4	4	3
IQR	1	1	1	1	1	1	1	1	1	1	1	1
Final questionnaire	Item 1	Item 2	Item 3	Item 4	Item 5	Item 6	Item 7	Item 8	Item 9	Item 10	Item 11	Item 12
Median	5	4	5	5	4	5	4	5	4	5	4	5
IQR	1	1	1	1	1	1	1	1	1	1	1	1

**Table 4:** Descriptive statistics of the questionnaire dimensions.

	ATG_S1	ATG_T1	GI_S1	GI_T1	ATG_S2	ATG_T2	GI_S2	GI_T2
Median	11	12	11	11	13	13	13	14
IQR	3	4	3	4	2	2	2	2

Note: Initial questionnaire (1), final questionnaire (2).

**Table 5:** Influence of clinical simulation on group cohesion—items.

	Z *	*p*-Value *	Size of Effect **
Item 1_1–Item 1_2	−9.935	0.000	0.563
Item 2_1–Item 2_2	−7.365	0.000	0.418
Item 3_1–Item 3_2	−11.393	0.000	0.646
Item 4_1–Item 4_2	−8.006	0.000	0.454
Item 5_1–Item 5_2	−4.483	0.000	0.254
Item 6_1–Item 6_2	−9.800	0.000	0.556
Item 7_1–Item 7_2	−6.401	0.000	0.363
Item 8_1–Item 8_2	−10.208	0.000	0.579
Item 9_1–Item 9_2	−7.059	0.000	0.400
Item 10_1–Item 10_2	−8.117	0.000	0.460
Item 11_1–Item 11_2	−6.954	0.000	0.394
Item 12_1–Item 12_2	−10.337	0.000	0.586

Note. * Wilcoxon test, Negative Z value: final questionnaire score (2) > initial questionnaire score (1). ** Rosenthal’s r, N = 311.

**Table 6:** Influence of clinical simulation on group cohesion—dimensions and total score.

	Z *	*p*-Value *	Size of Effect **
ATG-S_1–ATG-S_2	−10.549	0.000	0.598
ATG-T_1–ATG-T_2	−9.599	0.000	0.544
GI-S_1–GI-S_2	−9.805	0.000	0.556
GI-T_1–GI-T_2	−11.099	0.000	0.629
Total score_1–Total score_2	−12.046	0.000	0.683

Note. * Wilcoxon test, Negative Z value: final questionnaire score (2) > initial questionnaire score (1). ** Rosenthal’s r, N = 311.

## Data Availability

The data used to support the findings of this study are available from the corresponding author upon request.
